# A prospective comparison of UroVysion FISH and urine cytology in bladder cancer detection

**DOI:** 10.1186/s12885-017-3227-3

**Published:** 2017-04-07

**Authors:** Hugh J. Lavery, Boriana Zaharieva, Andrew McFaddin, Nyla Heerema, Kamal S. Pohar

**Affiliations:** 1 Department of Urology, Ohio State University Wexner Medical Center, Columbus, OH 43210 USA; 2 Comprehensive Cancer Center, The Ohio State University, Columbus, OH 43210 USA; 3 Department of Pathology, Division of Cytogenetics, Ohio State University Wexner Medical Center, Columbus, OH 43210 USA

**Keywords:** Urinary bladder neoplasm, In situ hybridization, Fluorescence, Cytology

## Abstract

**Background:**

UroVysion fluorescence in situ hybridization (uFISH) was reported to have superior sensitivity to urine cytology. However uFISH studies are limited by varying definitions of what is considered a positive result, absence of histopathology and small sample size. The aim of our study was to better determine the performance characteristics of uFISH and urine cytology by overcoming some of the deficiencies of the current literature.

**Methods:**

Intraoperative bladder wash cytology and uFISH were collected prospectively on all patients. Strict definitions for positivity of uFISH and cytology were determined before initiating the study. A re-review of false-negative uFISH specimens was performed to analyze potential sources of error. Sixteen bladder tumors embedded in paraffin were analyzed by uFISH and compared with the result in the urine.

**Results:**

One hundred and twenty-nine specimens were analyzed. Sensitivity was 67% and 69% (*p* = 0.54); specificity was 72% and 76% (*p* = 1.0), for uFISH and cytology, respectively. Thirty-two false negative uFISH samples were re-reviewed. Low grade tumors often showed cells with abnormal morphology and patchy DAPI staining but diploid chromosomal counts and a few high grade tumors had tetraploid counts but less than needed to interpret uFISH as positive. uFISH study of the tumors revealed three categories; positive in both tumor and urine (9), negative in both tumor and urine (5) and positive in tumor but negative in urine (2).

**Conclusion:**

In a pathologically-confirmed analysis of bladder washed urine specimens, uFISH does not outperform urine cytology in cancer detection.

## Background

UroVysion fluorescence in situ hybridization (uFISH) is a multitarget, multicolor FISH assay that has been developed for the detection of urothelial carcinoma in the urine [1]. The assay is mostly used in the surveillance of patients with a history of bladder cancer [2–4]. Numerous studies have compared the performance characteristics (sensitivity and specificity) of uFISH to urinary cytology [5–12]. A recent meta-analysis of the published literature on uFISH reported superior sensitivity and comparable specificity when compared to cytology [13].

uFISH is reported dichotomously (either positive or negative) based on criteria that include not only chromosomal changes but cell morphology. Despite the stringency of test reporting as either positive or negative, difficulty of interpretation of chromosomal and morphologic changes does exist, potentially influencing the usefulness of the test in cancer detection. Limitations of previous publications on uFISH include varying definitions of what is considered a positive result, lack of histopathology and small sample size [14–19]. Also the likelihood a given bladder cancer may not have the chromosomal alterations measured by uFISH and thus not capable of being detected by the test is not entirely known.

Similar to uFISH, urinary cytology is reported as positive or negative however additional terminology in reporting cytology specimens includes suspicious and atypical. The clinical significance of an atypical cytology has been debated and whether an atypical cytology is considered positive or negative influences the sensitivity of urine cytology in bladder cancer detection. A recent large scale study provides further evidence that classifying an atypical cytology as negative has little influence of the sensitivity of cancer detection [20].

The aim of our study was to better determine the performance characteristics of uFISH and urine cytology by overcoming some of the deficiencies of the current literature.

## Methods

### Sample collection

Institutional review board approval was obtained for an institutional tissue- and body fluid banking protocol. Patients were consented to allow for collection of urine specimens and fresh frozen and formalin-fixed paraffin embedded tumor tissue for research purposes, if not clinically needed, in a prospective manner on all patients undergoing transurethral resection of bladder tumor (TURBT) or radical cystectomy (RC). A total of 129 urine specimens were included in the study. Following induction of anesthesia and immediately prior to the surgical procedure, two 60 ml bladder-washed aliquots of urine were obtained via a rigid cystoscope for TURBT or urethral catheter for RC. The two aliquots were randomly assigned for urine cytology or uFISH analysis.

In addition, 16 formalin fixed paraffin embedded (FFPE) bladder tumor specimens and 4 normal bladder urothelial samples were analyzed by uFISH. A representative area containing ≥80% tumor cells was selected for uFISH testing on each FFPE slide. All tumors evaluated in this study represented primary bladder cancers.

### uFISH and cytology

All cytology specimens were analyzed by two ctyopathologists each with over 10 years of experience in urine cytology. Samples for conventional urine cytology were centrifuged and obtained by the Cytospin method. The slides were then stained by the standard Papanicolaou method. All uFISH specimens were processed according to the manufacturer’s protocol by one cytogenetic technician and analyzed by one cytogeneticist with many years of experience in cytogenetics and cell morphology. The pathologists and cytogeneticist had no prior knowledge of this study. Tissue samples were evaluated by standard institutional protocol.

uFISH positivity in the bladder washings was determined by the criteria as listed in the package insert after all cells in a 15 mm diameter site were evaluated. Four or more abnormal cells that had chromosomal gain for at least two of chromosomes 3, 7 or 17 or ≥12 cells with homozygous loss of 9p21 or samples with ≥10 tetraploid cells with normal morphology were considered positive.

A previous unpublished institutional effort determined that urothelial cancer was proven histologically when the cytology was reported as positive in 9/9 (100%), suspicious in 8/9 (89%) and atypical in 1/13 (7%) patients. Importantly all patients with a negative cytology all had a normal cystoscopy. Therefore for the purpose of this study a positive or suspicious cytology was considered positive and an atypical and negative cytology was considered negative. These definitions were generated prior to data analysis.

All TURBT were performed in a standardized manner that included an initial assessment of the entire bladder and prostatic urothelium and a pre- and post-resection exam under anesthesia. All areas of visible tumor as well as sites of previous resection were resected in their entirety. Random bladder and/or prostate urothelial biopsies were frequently performed. Upper urinary tract evaluation by urography was always performed and the presence of upper urinary tract carcinomas was excluded. Radical cystectomy was performed as previously reported [21]. Final histopathology from the operative specimen was considered the standard to which uFISH and cytology were compared.

At the completion of the study the cytogeneticist was unblinded and a post-hoc analysis of false-positive and false-negative uFISH was conducted. Information regarding cellularity, morphology, DAPI (4,6-Diamidino-2-Phenylindole) staining, and aneuploidy of the uFISH chromosomes that was not presented in the initial report was collected. All false-negative uFISH slides were re-run with residual urine from the original aliquot to ensure quality control.

### uFISH in the tumors

For FISH, 5-μm paraffin sections were placed in xylene for 30 (3 × 10) minutes followed by dehydration in 100% ethanol for 10 (2 × 5) minutes. Prior to hybridization, the slides were treated with Paraffin Pretreatment Reagent Kit (Abbott Molecular Inc., Des Plaines, IL, USA, Cat. # 32–801,200) and dehydrated in 70%, 85%, and 100% alcohol solutions for 5 min each. FISH was performed using UroVysion probe (Abbott Molecular, Des Plaines, IL). Denaturation of the DNA was carried out at 75 °C for 10 min (probe mixture) or 5 min (slides). The probe mixture was applied to the slides and hybridized overnight in a moist chamber at 37 °C. The post-hybridization washes were performed as described in ‘LSI procedure’ (Abbott Molecular Inc., Des Plaines, IL, USA). Slides were counterstained with 125 ng/ml DAPI in antifade. A minimum of 25 tumor cells were analyzed for copy number changes of chromosomes 3, 7, 17 and 9p21 [22]. If no abnormalities were detected, the remaining cells were counted until a sufficient number of cells with chromosomal abnormalities were found, or until 200 cells were evaluated, or the whole specimen was analysed. A positive result was the presence of ≥4 cells with gains of two or more of chromosomes 3, 7 and 17. In the case of chromosome 9p21, a positive result was considered when ≥12 cells showed absence of 9p21 signals.

### Statistics

Data was collected and compiled using SPSS v.15.0. Statistical analyses were performed using McNemar’s test for comparisons between uFISH and cytology and the chi-squared test for other comparisons. All *p*-values are two-sided.

## Results

One hundred and twenty-nine urine samples were included in the study and collected at the time of TURBT (*n* = 94) or at RC (*n* = 35). Baseline characteristics of the study population and TURBT or RC pathology are listed in Table 1.

**Table 1:** Patient and tumor characteristics

Mean Age (yrs)	68
Male:Female	102:27
TURBT	94 (73%)
Radical Cystectomy	35 (27%)
Indication for TURBT:	
Cystoscopic Abnormality:	48 (51%)
New diagnosis	19
Recurrence	29
Completion of initial TURBT	38 (40%)
Isolated positive cytology	8 (9%)
Tumor grade	
Low	14 (14%)
High	87 (86%)
Tumor stage	
T0	28 (22%)
Ta	31 (24%)
CIS alone	16 (12%)
T1	19 (15%)
T2	17 (13%)
T3	15 (12%)
T4	3 (2%)
Any CIS	47 (36%)

The indication for TURBT was suspicious cystoscopic findings for cancer in 48 (51%) patients on surveillance for a history of non-muscle invasive bladder cancer (NMIBC), repeat TURBT within 6 weeks of the initial TURBT for confirming completeness of tumor resection in 38 (40%) patients [23] and a positive urinary cytology with normal cystoscopic findings in 8 (9%) patients. Eighty percent of the TURBTs were performed on patients who had previously been diagnosed with bladder cancer and 73 (78%) specimens collected by TURBT identified the presence of cancer. All of the RC specimens had evidence for cancer except one specimen. A histologic diagnosis of cancer was present in 101 specimens (78%).

Urine cytologies were reported as positive, consistent or suspicious for carcinoma or reported as negative, atypical or the presence of papillary clusters without fibrovascular cores. For the purpose of this study the definition of a positive or negative cytology is given in Table 2 along with the relative frequency of the cytologic descriptions. The interpretation of uFISH is described in the methods.

**Table 2:** Nomenclature for classification of urinary cytology reports

Positive cytology (*n* = 76)
Urothelial Carcinoma/Positive for malignancy (40) Severely atypical cells consistent with high grade urothelial carcinoma (6) Suspicious for carcinoma (30)
Negative cytology (*n* = 53)
No evidence of carcinoma/malignancy (32) Urothelial atypia (18) Papillary clusters without fibrovascular cores (3)

The overall performance of uFISH and cytology is shown in Table 3. uFISH was positive in 73 (56%) collected specimens and cytology was positive in 76 (59%). Overall sensitivity was 67% and 69% for uFISH and cytology (*p* = 0.54) and specificity was 72% and 76% for uFISH and cytology (*p* = 1.0), respectively. Sensitivity of the tests by tumor grade and stage is given in Table 4. Each of the sensitivity comparisons between uFISH and cytology was not statistically significant (*p* > 0.05). Similarly, no significant differences in sensitivity between uFISH and cytology were found when restricting the analysis to TURBT or RC procedures.

**Table 3:** uFISH and cytology performance by stage

Pathological stage		Negative uFISH	Positive uFISH	Negative cytology	Positive cytology
No Tumor	28	21	7	22	6
Tumor	101^a^	32	66	31	70
• Non-muscle invasive bladder cancer (≤ pT1)	66^a^	23	40	22	44
• Muscle invasive bladder cancer (≥ pT2)	35	9	26	9	26

^a^Three samples were not analyzed by uFISH due to lack of enough cellular material

**Table 4:** Sensitivity of uFISH and cytology by grade and stage

	n^a^	uFISH sensitivity (%)	Cytology sensitivity (%)	*p*-value*	False negative uFISH (*n* = 32)	False negative cytology (*n* = 31)
Low Grade	14	25%	36%	0.63	9	9
High Grade	87	73%	75%	0.82	23	22
Any CIS	47	79%	83%	0.73	10	8
CIS alone	16	75%	81%	1.0	4	3
≤pT1	66^a^	63%	67%	0.45	23	22
≥ pT2	35	74%	74%	1.0	9	9

^a^Three samples were not analyzed by uFISH due to lack of enough cellular material

*Comparison of uFISH and Cytology sensitivity

The grade and stage of the tumors of the 32 false-negative uFISH specimens and 31 false negative cytologies are also presented in Table 4. False negative results were noted in both low and high grade tumors as well as tumors of all pathologic stages, including carcinoma in situ. Specific analysis of the low grade Ta tumors identified a false negative result in 9 uFISH and 9 cytology specimens. Re-review of the uFISH slides from the false negative specimens often showed the presence of cells with abnormal morphology and patchy DAPI staining but diploid chromosomal counts and enough cellular material to interpret the test result as negative. In addition, of the 32 false-negative uFISH specimens, nine were from RC specimens. Each of the 9 RC specimens had significant papillary tumor burden on gross and microscopic pathology and final pathologic stage was pT2 (4), pT3 (4) and pT4 (1). Some of these same RC specimens also had the presence of carcinoma in situ (CIS). Re-review of the uFISH slides from these 9 specimens demonstrated three tumors with tetraploid counts but less than the number needed to interpret FISH as positive.

Analysis of the complementarity of the two tests was performed by examining the number of false-negatives from one test that were detected by the other. Fourteen (44%) false-negative uFISH had positive cytologies, while 10 (36%) false-negative cytologies had positive uFISH. Thus 64% of tumors missed by cytology would also have been missed by a concomitant uFISH. The sensitivity when either uFISH or cytology is considered positive was 82%, while the specificity of such a definition was 59%. If uFISH and cytology are both positive concurrently, the sensitivity decreases to 57% while specificity is 93%.

Review of the longterm clinical history of the 7 false-positive uFISH specimens revealed that three patients later underwent RC and another patient was recommended to undergo a RC for high risk of disease progression. Each of these four patients had muscle invasive disease with or without CIS when they recurred. Two additional patients developed upper urinary tract urothelial cancer and underwent a nephroureterectomy. The remaining patient has not recurred in more than two years of follow-up.

Four normal FFPE bladder urothelial samples and 16 FFPE tumor samples were evaluated by FISH to determine how often the chromosomal changes quantitated by the UroVysion FISH assay were present in the tumor. About 20% of the low and high grade tumors and 20% of the NMIBC and MIBC included in the study were chosen to measure UroVysion FISH positivity. All four normal samples analyzed were uFISH negative because of normal copy number counts. The uFISH data for both the tumor and the corresponding urine specimen are presented in Table 5. Importantly 5 of 16 tumors (31%) were uFISH negative and the corresponding urine sample for these tumors was also uFISH negative. The uFISH negative tumors included tumors of low and high grade as well as superficial and invasive disease. Also two tumors determined to be uFISH positive had a corresponding uFISH negative urine sample, including one high grade tumor.

**Table 5:** uFISH results in formalin-fixed paraffin embedded (FFPE) tumors and corresponding urine samples

Tumors		uFISH negative in FFPE tumors	uFISH negative in urine samples	uFISH positive in FFPE tumors	uFISH positive in urine samples
Low Grade	2	1	2	1	0
High Grade	14	4	5	10	9
≤pT1	9	1	2	8	7
≥ pT2	7	4	5	3	2

## Discussion

The main reported advantage of uFISH is increased sensitivity of cancer detection when compared to urine cytology, especially in high grade tumors [18, 24]. However, false-negative results still occur and may be due to failure of tumor detection at cystoscopy or an “anticipatory positive” UroVysion FISH [25]. In addition, in order for uFISH to identify the presence of a cancer it is necessary the cancer has gains of at least two of chromosomes 3, 7 or 17 in at least four or loss of 9p21 in at least twelve, morphologically abnormal cells, in accordance with the FDA approval of the uFISH assay [1, 5]. And in fact, our study suggests not all bladder cancers have the chromosomal alterations detected by uFISH.

In addition, the uFISH assay is also associated with false positive results. One explanation for false positive results is the presence of tetraploidy in the uFISH assay that may reflect normal cell division or a proliferative response of the bladder epithelium to an inflammatory insult and not a cancerous process. Zellweger et al. determined the number of uFISH false positives can be reduced by establishing the presence of tetraploidy in at least 10 morphologically abnormal cells as this is more reflective of the presence of cancer [26]. The importance of defining the number of cells with tetraploidy in the uFISH assay was further supported by the work of Kipp et al. [27]. Patients in both studies with a normal cystoscopy and a positive uFISH result based on defining teteraploidy in at least 10 cells were more likely to be diagnosed with cancer at the time of the original bladder biopsy or subsequent bladder biopsy thus improving upon both the sensitivity and specificity of the uFISH assay. Based on these publications our institution and others include tetraploidy in at least 10 morphologically abnormal cells in the definition of a positive uFISH result, in addition to the FDA approved criteria [27].

Our discussion mostly focuses on the false negative result and sensitivity of the uFISH assay. An extensive review by Jones of nine uFISH studies reported a sensitivity of 96% in CIS lesions (44 specimens) and 94% in 87 invasive tumor specimens [28]. Promotional literature for the uFISH test kit reports a “near 100% sensitivity for dangerous high grade lesions” [17]. However our study findings are not consistent with these reports as we found a sensitivity of 79% in 47 CIS lesions and 74% in 35 invasive lesions. Moreover, our study findings are similar to other studies that indicate the performance of traditional urine cytology is similar to uFISH in bladder cancer detection [29, 30].

Why do our results of uFISH sensitivity differ from some other reports? Our study attempted to reduce the limitations of previous publications that may have included varying definitions of what is considered a positive uFISH result, lack of histopathology, heterogeneous patient population and small sample size. In our study all patients were evaluated in the operating room for presumed bladder cancer and had concurrent bladder washed urine samples thus increasing the cellularity of the specimen and histopathology for comparison. Only 14% of patients had low-grade tumors and 27% of specimens were acquired at the time of radical cystectomy for invasive disease. Therefore the patients included in the study presumably have a high likelihood of a positive uFISH and are more representative of the true performance characteristics of uFISH.

After the study concluded we validated our uFISH sensitivity analysis by re-evaluating the false negative cases. To this end a single experienced cytogenetic technician repeated the analysis of all false-negative specimens from stored residual urine and compared the result to the originally processed urine sample for the same patient. All false negative uFISH urine samples were confirmed to be negative on repeat evaluation. Importantly several samples contained morphologically abnormal cells and tetraploidy in less than 10 cells of the targeted probes and absence of the FDA approved criteria for a positive test result. We also recognized the urine samples obtained from most low grade tumors were comprised of morphologically abnormal cells with patchy DAPI nuclear staining but had normal diploid counts of the Urovysion markers. Therefore it is possible that bladder tumors with a negative urine uFISH do not have the chromosomal alterations that the uFISH test is detecting.

To investigate this possibility we directly performed uFISH analysis of 16 bladder tumors of varying grades and stages. Our findings determined that about 30% of bladder tumors do not have aneuploidy of chromosomes 3, 7, 17 or 9p21 and may explain some of the uFISH false negatives in our evaluation of the urine samples in not only high grade tumors but also low grade tumors. However, our study determined that all tumors, with two exceptions, with the uFISH chromosomal alterations can be detected in the corresponding urine sample. The findings of our study suggest the need for a more comprehensive cytogenetic evaluation of bladder cancer is needed to improve FISH based testing. The benefit of using complementary molecular tests in the urine such as loss of heterozygosity, nuclear matrix protein 22 (NMP22) and array comparative genomic hybridization (array CGH) has been shown in the literature [31–33]. Therefore, as the cost of next-generation sequencing, microarray-based CGH and other global approaches decreases and becomes more clinically applicable these technologies should be studied alone or as a complement to uFISH to improve bladder cancer detection.

As a final comment, in our study the sensitivity of urine cytology was much higher in high grade tumors when compared to low grade tumors. This was an expected result however we did note a higher sensitivity (36%) for urine cytology in low grade tumors in our study when compared to prior publications. The likely explanation of a positive cytology in the six patients with low grade tumors in this study is the fact that five of the patients were defined as intermediate risk tumors characterized by multifocality and tumors were greater than 3 cm in size. The collection of the urine samples by washing or barbotage is known to increase cellular yield of the sample. Therefore when the burden of tumor is high, even in the context of low grade tumors, it is more likely a positive cytology will be obtained when the specimen is collected by barbotage compared to a voided sample.

Our study design is clearly limited by its lack of clinical applicability. However, the study was purposely designed to advance the current state of knowledge of uFISH by determining its true performance characteristics as it is used today and to recognize opportunities for improvement. The study provides valuable new insights on improving FISH based assays and the need to better standardize definitions of reporting a positive result.

## Conclusions

In a prospective, pathologically-confirmed analysis of intraoperative bladder washed urine specimens, Urovysion FISH does not outperform traditional urine cytology in either sensitivity or specificity.

## Abbreviations

CGH: Comparative genomic hybridization
CIS: Carcinoma in situ
DAPI: 4,6-Diamidino-2-Phenylindole
LSI: Locus Specific Identifier
NMP22: nuclear matrix protein 22
OR: Operating room
RC: Radical cystectomy
TURBT: Transurethral resection of bladder tumor
uFISH: UroVysion fluorescence in situ hybridization
